# The link of universalism, transformational leadership, innovativeness, and leader effectiveness: a multivariate dataset

**DOI:** 10.3389/fpsyg.2023.1181844

**Published:** 2024-01-17

**Authors:** Sunu Widianto, Budi Harsanto, Arviansyah Arviansyah

**Affiliations:** ^1^Department of Management and Business, Padjadjaran University, Bandung, Indonesia; ^2^Department of Management, University of Indonesia, Depok, West Java, Indonesia

**Keywords:** universalism, transformational leadership, innovativeness, leader effectiveness, SMEs

## Introduction

The role of ethics and morals in excellent leaders has been reemphasized by leadership and management theorists (George, 2003; Luthans and Avolio, 2003; May et al., 2003; Avolio and Gardner, 2005; Brown and Treviño, 2006; Hoch et al., 2018). Leader value has become an emerging topic among practitioners and academia (Newstead et al., 2021). De Hoogh and Den Hartog (2008) investigated the relationship between leaders' social responsibility and the ethical aspects of leadership, such as morality and fairness, role clarification, and power sharing. De Hoogh and Den Hartog (2008) found that leaders with high social responsibility have a high level of ethical values and ethical conduct (Banks et al., 2016; Gardner and Wickramasinghe, 2023). Value is generally used to understand the characteristics and behavior of a community or individual. Value becomes a crucial discussion in social sciences as it constitutes what is good or bad, fair, or unfair, and legitimate or illegitimate in the life of a community (Boudon, 2001; Newstead et al., 2021). Universalism is one of the value aspects of human or work values. According to Schwartz (2006), universalism is a value of understanding, appreciation, tolerance, and protection devoted to the welfare of people and nature. Universalism reflects inner harmony in everyday life, which can be found in diverse people from different parts of the world. Nevertheless, although some value leadership styles, such as ethical leadership, authentic leadership, and transformational leadership, have shown a link to desired outcomes (i.e., performance and effectiveness) (Hoch et al., 2018), how the mechanism of value links to leadership style remain scant. effectiveness) (Ng, 2017), yet few studies have investigated the link between values and transformational leadership toward desired outcomes such as innovation and leader effectiveness.

Innovation in current business is a strategic issue that becomes an essential element to survive and grow in a competitive, dynamic, and turbulent atmosphere. Zahra and Covin (1994) conducted research on 120 strategic business units and found that the differences in strategies associated with different types of innovation will ultimately affect the firm financial performance. The concept of transformational leadership was first introduced by Burns in 1978. Many decades later, the interest in this area continues to grow until now. Kuhnert and Lewis (1987) developed a two-level transformational leadership and three- stage developmental model of leadership. Previous studies have endeavored to connect transformational leadership with other aspects of the organization. Jung et al. (2003) indicated that transformational leadership was positively related to organizational empowerment and innovation climate. Peterson et al. (2009) made a comparison between start-ups and established firms. They argued that transformational leadership was more strongly connected to start-up performance than established companies. In the Asian context, Harsanto and Roelfsema (2015) found a strong effect of transformational leadership on sales growth mediated by entrepreneurial orientations. Moreover, in line with Derue et al. (2011)'s call for integration across leadership forms, the current study intends not only to comprehend but also to analyze the three ethical/moral values-based leadership forms in the context of transformational leadership (Garg and Krishnan, 2003; Copeland, 2014; Abay et al., 2023). There has been some debate about whether these morally focused leadership techniques are conceptually separate from transformative leadership (e.g., Avolio and Gardner, 2005).

Extant research has provided some empirical evidence of significant associations between transformational leadership and ethical (Ng and Feldman, 2015), authentic (Riggio et al., 2010), and servant leadership (Van Dierendonck et al., 2014). Prior studies examining the link between ethical value, leadership, and business performance have been studied by several scholars (Siangchokyoo et al., 2020). However, how human or work value is linked to leadership style, innovativeness, and effectiveness is rarely elaborated (Li et al., 2016; Cho and Kao, 2022). Thus, this paper aims to examine the relationship between universalism, transformational leadership, innovativeness, and leader effectiveness in the context of SMEs in Indonesia, which has the largest population and economy in Southeast Asia.

## Methods

### Sample

The study involved 100 owners and senior managers of small and medium organizations in Bandung, Indonesia. The owners/senior managers were selected because they are the key informants and are knowledgeable about the organizations' whole activities (Engelen et al., 2014). Moreover, we administered the questionnaires via an online survey to various types of businesses manufacturing. The main industries are textiles, food processing, and light manufacturing industries.

### Procedures

The data were collected via an online survey. Firstly, we began administering the online survey based on our sampling frame. Afterward, since some SMEs were not already operating their business, we asked the respondents participating in the online survey to kindly distribute the online survey to their communities and colleagues who also run SMEs.

### Measures

We measured transformational leadership, universalism, and leader effectiveness using well-established measurements on Likert-type scales from 0 (not at all) to 5 (Frequently, if not always) (see Appendix).

#### Universalism

We used items from Schwartz (2006) to measure organizational culture dimensions (three items).

#### Transformational leadership

We measured the construct using the MLQ developed by Bass and Steidlmeier (1999), which comprises five dimensions (20 items): idealized attributes, idealized behaviors, inspirational motivation, intellectual stimulation, and individual consideration.

#### Innovativeness

We used innovation growth by analyzing the innovation performance for the last 3 years. This innovation performance includes three questions: whether the companies introduce new products or services, what percentages of annual sales are accounted for the new products or services, and whether the companies introduce new processes.

#### Leader effectiveness (four items)

We used the leadership outcomes from MLQ apart from extra effort and satisfaction developed by Bass and Steidlmeier (1999). Effectiveness is measured by how effectively the leaders achieve the organization's objectives, meet job expectations, represent others to higher authorities, and lead groups.

## Results

### Descriptive statistics

The correlations are presented in Table 1 indicating the relations between variables.

**Table 1:** Descriptive statistics and correlations.

	***N***	**SD**	**Mean**	**1**	**2**	**3**
Universalism	100	0.71	4.01			
Transformational leadership	100	0.59	4.13	0.48^**^		
Innovativeness	100	0.85	3.82	0.39^**^	0.56^**^	
Leader effectiveness	100	0.66	3.87	0.27^**^	0.56^**^	0.55^**^

^**^Correlation is significant at the 0.01 level (2-tailed).

#### Measurement fit model: validity, reliability, fit indices

Before testing the model, we examined the instruments' validity and reliability. We employed a partial least square (PLS) algorithm in SmartPLS 3 to examine the loading of item. Partial least square were used since it can test a relatively small sample and provide the factor loading for each item. Thus, the partial least square is fit for our study. Based on the analysis of variance extracted (AVE) criterion for divergent and affirm that each latent construct (see Table 2). Furthermore, we also test the model fit of the model. Model fit indices indicate that all index parameters fall into the acceptable category (see Table 3) (Henseler et al., 2015).

**Table 2:** Heterotrait-Monotrait (HTMT).

	**Innovation**	**Leader effectiveness**	**Transformational leadership**	**Universalism**
**Innovation**				
Leader effectiveness	0.673			
Transformational leadership	0.642	0.661		
Universalism	0.538	0.389	0.639	

**Table 3:** Model Fit indices, composite reliability, and AVE.

**Model**	**Chi-Square**	**NFI**	**D-G**	**D-ULS**	**SRMR**
CFA model	416.187	0.709	0.805	2.143	0.085
Recommended value	>chi-square table (Hair et al., 2017)	Closer to 1 (Bentler and Bonett, 1980)	< Interval confidence level (Hair et al., 2017)	< Interval confidence level (Hair et al., 2017)	< 0.10 (Hu and Bentler, 1999)
25-1,15498pt**Variable name**	**No of items**		**Composite reliability**	**AVE**	
Universalism	3		0.806	0.583	
Transformational leadership	20		0.935	0.510	
Innovativeness	3		0.910	0.771	
Leader effectiveness	4		0.866	0.618	

#### Model testing

**Figure 1:**
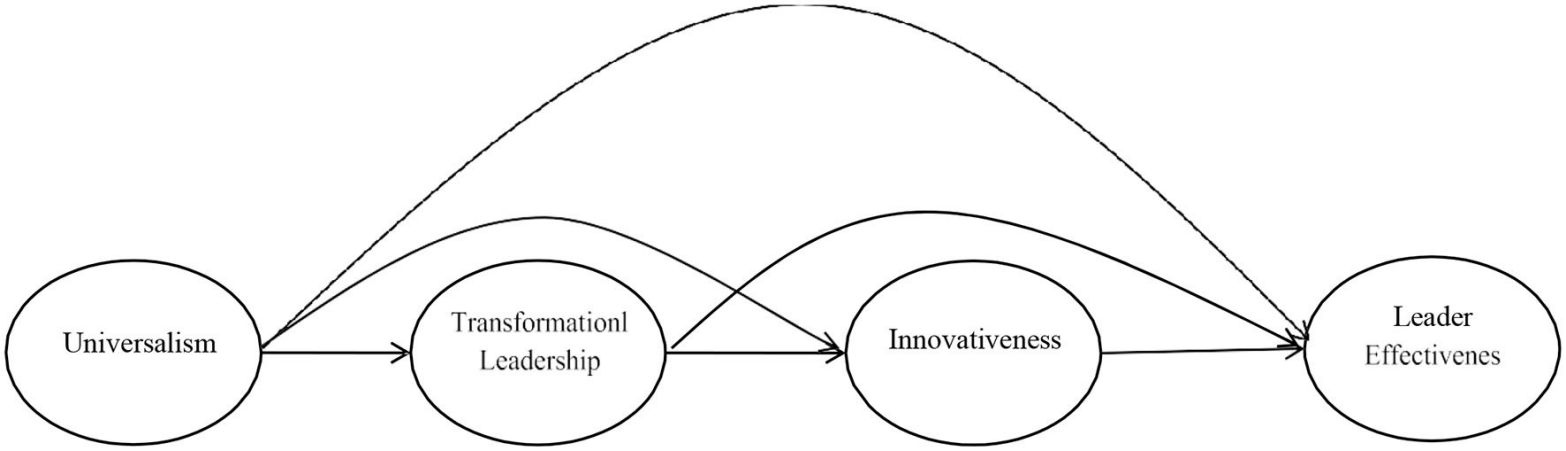
The conceptual framework.

To test the mediating effect, a three-path mediation model (Figure 1) was tested. In such a model, two mediators (transformational leadership and innovativeness) intervene in a series between an independent and a dependent variable (X and Y). Taylor et al. (2008) indicated that three conditions need to be fulfilled to conclude that such a model is supported: (1) the relationship between X and M1 is significant, (2) the relationship between M1 and M2, while controlling for X, is significant, and (3) the relationship between M2 and Y, while controlling for X and M1, is significant. We therefore test the three-path mediation model by employed PLS Bootstrapping. The results (i.e., T-statistics and *p*-value) shows the universalism relates to transformational leadership (β = 6.49, *p* < 0.00) while universalism does not link to innovation (β = 1.49, *p* < ns) and leader effectiveness (β = 0.56, *P* < ns). In addition, transformational leadership is link to innovativeness (β = 4.31, *p* < 0.01) and leader effectiveness (β = 2.82, *p* < 0.01) whereas the link between innovation and leader effectiveness is significant (β = 2.16, *p* < 0.01). Based on Taylor et al. (2008) criteria, it indicates that the three-path mediation model meet the criteria (see Table 4).

**Table 4:** Results of the link of conceptual framework.

	**Original sample (O)**	**Sample mean (M)**	**Standard deviation (STDEV)**	***T*-statistics (|O/STDEV|)**	***P*-values**
Innovation -> leader effectiveness	0.331	0.32	0.153	2.168	0.031
Transformational leadership -> innovation	0.505	0.497	0.117	4.317	0.000
Transformational leadership -> leader effectiveness	0.438	0.438	0.155	2.82	0.005
Universalism -> innovation	0.144	0.144	0.096	1.494	0.136
Universalism -> leader effectiveness	−0.057	−0.046	0.101	0.56	0.575
Universalism -> transformational leadership	0.518	0.543	0.08	6.49	0.000

## Discussion

This study provides one-of-a-kind primary survey-based data to comprehend the association of organizational factors (viz. leader value, leadership style) and contextual factors (viz. universalism, transformational leadership, and leader effectiveness) on SMEs' innovation in Indonesia. Most previous studies on transformational leadership style have only focused on the consequences of transformational leadership style, such as job performance and work engagement. Therefore, this study investigates not only the consequences of transformational leadership but also the human or work value factor (i.e., universalism) as an antecedent of transformational leadership, which is extremely rare in the literature on transformational leadership (Jin et al., 2016; Lin et al., 2022; Shamshad and Naqi Khan, 2022). These data, which include information on universalism, leadership, innovation, and leader effectiveness, can be helpful for researchers researching antecedents of value-based leadership and innovation as well as leader effectiveness. Moreover, the findings from this data can also be of practical use for the SMEs and policymakers in leadership development of the owners or senior management of SMEs to enhance their leadership effectiveness. The data can be used for advanced level modeling such as mediation, moderation, and moderated mediation because they are metric/quantitative in nature and have been collected using reflecting scales (Ng, 2017).

Furthermore, this study contributes to the existing body of knowledge in leadership and organizational studies in three significant ways. Firstly, it establishes a novel connection between value-based leadership and innovation within a unified model, a gap that has been inadequately addressed in the literature (Snyder et al., 2017; Hunsaker, 2022). The absence of a simultaneous examination of transformational leadership and innovativeness as subsequent mediators leaves our understanding of the impact of value-based leadership, particularly in terms of universalism, on leader effectiveness unclear. As a result, managerial initiatives aimed at cultivating effective leadership may be rendered unjustifiable if not grounded in a comprehensive assessment. For instance, leaders seeking to enhance their effectiveness may inadvertently focus solely on fostering innovativeness without considering the crucial elements of inspiring motivation and individualized consideration associated with transformational leadership.

Secondly, this study enriches the literature by shedding light on the antecedents of transformational leadership, an aspect that has received relatively limited attention (Morf and Bakker, 2022). While prior research has predominantly explored the direct effects of transformational leadership on desired outcomes (Hoch et al., 2018), the investigation into how a leader's values interact with leadership styles to ultimately influence leader effectiveness has been understudied. Despite literature acknowledging that certain leadership styles cultivate innovativeness and leader effectiveness, this study adopts a social exchange lens. It highlights how a leader's values, specifically universalism, justify the emergence of innovativeness and contribute to the perception of leader effectiveness.

Thirdly, by integrating value-based leadership with the social exchange theory, this study enhances our comprehension of leader effectiveness. It provides a more comprehensive explanation of how a leader's values, in conjunction with social exchange dynamics, contribute to the overall effectiveness of leadership in an organizational context.

## Practical implications

The study highlights the importance of universalism leadership toward leader effectiveness. Organizations should promote universalism value among leaders and provide training and development programs in order to cultivate transformational leadership skills. Transformational leadership may be deemed insufficient due to the absence of a strong, explicit moral dimension (Hoch et al., 2018). Thus, to stimulate innovativeness and leader effectiveness, leaders not only should apply transformational leadership style but also embrace universalism. Universalism values, such as understanding, appreciation, tolerance, and protection of people and nature, should be developed into transformational leadership style. Transformational leaders should encourage a sense of social responsibility and promote inclusivity, diversity, and sustainability within their organizations. By flourishing universalism values, organizations can benefit innovativeness and leader effectiveness. Organizations should identify and develop transformational leaders who inspire and motivate their teams, stimulate intellectual curiosity, and provide individualized support. By nurturing transformational leadership, organizations not only can drive innovation yet ultimately will see as effective leader.

Innovation is crucial for organizational survival and growth. Therefore, organizations should create a supportive environment that encourages and rewards innovation. Leaders should promote a culture of continuous learning, experimentation, and risk-taking. By fostering a climate that values and supports innovation, organizations can stay competitive and adapt to changing market dynamics.

## Future research directions

Future research could explore the mediating mechanisms through which universalism values influence transformational leadership and its outcomes (Ng, 2017). This could provide a deeper understanding of the underlying processes and shed light on the specific pathways through which values impact leadership effectiveness and innovation. Moderation analysis: Investigating potential moderators of the relationships between universalism, transformational leadership, and outcomes could offer valuable insights. Factors such as organizational culture, industry context, or individual characteristics may moderate the relationships, and exploring these interactions can provide a more nuanced understanding of the dynamics at play. Longitudinal studies: Conducting longitudinal studies would allow for the examination of the causal relationships between universalism values, transformational leadership, innovation, and leader effectiveness over time. Longitudinal designs can provide more robust evidence and capture the dynamic nature of these constructs. Cross-cultural studies: Replicating the study in different cultural contexts would contribute to our understanding of how universalism values and transformational leadership operate across diverse cultural settings. Comparing the findings across cultures can reveal cultural nuances and identify potential boundary conditions for the relationships studied. Mixed-methods approaches: Combining quantitative surveys with qualitative methods, such as interviews or case studies, can provide richer insights into the experiences and perceptions of leaders and employees. Moreover, scholars must also evaluate and refine key assumptions along the way to new paradigm consensus. Therefore, mixed- methodologies are most suited to this task. Mixed-method research concentrating on follower transformation, for example, might evaluate potential mediators found through inductive investigations, combining traditional quantitative approaches to hypothesis testing with qualitative interviews to open the black box of follower transformation (Siangchokyoo et al., 2020). This mixed-methods approach would help capture the complexity of the relationships and offer a more comprehensive understanding of the phenomenon under investigation.

The integration of various and applicable theories to assist explain previously or currently inexplicable phenomena and organize theoretical predictions is a feature of these types of investigations (intermediate studies; Edmondson and McManus, 2007). To explain why transformational leader behaviors result in positive follower psychological changes, scholars have previously recommended theories such as social learning theory (Bandura, 1977), social exchange theory (Graen and Uhl-Bien, 1995), and social identity theory (Ashforth and Mael, 1989). Moreover, opens up possibilities for using other existing ideas to further demonstrate how leaders impact followers such as self-regulation theory (Lord et al., 2010) and theories about emotions (e.g., affective events theory; Weiss and Cropanzano, 1996; event system theory; Morgeson et al., 2015).

In addition, future research might be explored most particularly, authentic leadership and servant leadership (Siangchokyoo et al., 2020). For intance, how value-based leadership or work values link to authentic leadership and servant leadership which results to innovative behavior of the follower and the effectiveness of these type of leadership.

## Data availability statement

The data that support the findings of this study are available from the corresponding author, [SW], upon reasonable request.

## Ethics statement

The studies involving humans were approved by the Committee of Ethics of Padjadjaran University. The studies were conducted in accordance with the local legislation and institutional requirements. The participants provided their written informed consent to participate in this study. Written informed consent was obtained from the individual(s) for the publication of any potentially identifiable images or data included in this article.

## Author contributions

All authors listed have made a substantial, direct, and intellectual contribution to the work and approved it for publication.

## Appendix 1

### Universalism

To what extent your immediate supervisor…

______1. She/he thinks it is important that every person in the world should be treated equally. She/he believes everyone should have equal opportunities in life.

______2. It is important to her/him to listen to people who are different from her/him. Even when she/he disagrees with them, she/he still wants to understand them

______3. She/he strongly believes that people should care for nature. Looking after environment is important to her/him.

### Transformational leadership Questionnaire

My immediate supervisor…

______1. Instills pride in me for being associated with him/her

______2. Goes beyond self-interest for the good of the group

______3. Acts in ways that builds my respect

______4. Displays a sense of power and confidence

______5. Talks about their most important values and beliefs

______6. Specifies the importance of having a strong sense of purpose

______7. Considers the moral and ethical consequences of decisions vadjust

______8. Emphasizes the importance of having a collective sense of mission

______9. Talks optimistically about the future

______10. Talks enthusiastically about what needs to be accomplished

______11. Articulates a compelling vision of the future

______12. Expresses confidence that goals will be achieved

______13. Re-examines critical assumptions to question whether they are appropriate

______14. Seeks differing perspectives when solving problems

______15. Gets me to look at problems from many different angles

______16. Suggests new ways of looking at how to complete assignments

______17. Spends time teaching and coaching

______18. Treats me as an individual rather than just as a member of a group

______19. Considers me as having different needs, abilities, and aspirations from others

______20. Helps me to develop my strengths

### Leader effectiveness

My immediate supervisor…

______1. Is effective in meeting my job-related needs

______2. Is effective in representing me to higher authority

______3. Is effective in meeting organizational requirements

______4. Leads a group that is effective
